# The Relationship Between Vitamin D, Clinical Manifestations, and Functional Network Connectivity in Female Patients With Major Depressive Disorder

**DOI:** 10.3389/fnagi.2022.817607

**Published:** 2022-02-10

**Authors:** Dao-min Zhu, Wenming Zhao, Shunshun Cui, Ping Jiang, Yu Zhang, Cun Zhang, Jiajia Zhu, Yongqiang Yu

**Affiliations:** ^1^Department of Radiology, The First Affiliated Hospital of Anhui Medical University, Hefei, China; ^2^Department of Sleep Disorders, Affiliated Psychological Hospital of Anhui Medical University, Hefei, China; ^3^Hefei Fourth People’s Hospital, Hefei, China; ^4^Anhui Mental Health Center, Hefei, China; ^5^Research Center of Clinical Medical Imaging, Anhui Province, Hefei, China; ^6^Anhui Provincial Institute of Translational Medicine, Hefei, China

**Keywords:** major depressive disorder, vitamin D, gender, functional network connectivity, functional MRI

## Abstract

Evidence suggests the pivotal role of vitamin D in the pathophysiology of major depressive disorder (MDD) via its effects on the brain. Gender differences exist in both depression and vitamin D level. Our objective was to investigate the association between gender, vitamin D, clinical manifestations, and functional network connectivity in a large sample of MDD patients and healthy controls. Resting-state functional MRI data were collected from 122 patients and 119 controls, with independent component analysis adopted to examine large-scale inter- and intranetwork functional connectivity. Serum concentration of vitamin D (SCVD) and clinical manifestations were also assessed. MDD patients exhibited lower SCVD than controls in females but not males. Moreover, we identified a female-specific association between lower SCVD and poorer cognitive performance. Concurrently, MDD-related functional network connectivity changes were correlated with SCVD in females as well as depression and anxiety symptoms in female patients. Remarkably, MDD- and SCVD-related functional network connectivity alterations mediated the associations between SCVD and cognition in females. Aside from providing evidence for a female-specific neurobiological mechanism whereby low vitamin D might contribute to MDD and its associated clinical characteristics, our findings inform a novel conceptualization that adjuvant vitamin D supplementation therapy may yield clinical benefits in improving treatment outcomes in female patients with MDD.

## Introduction

Major depressive disorder (MDD) is a prevalent major psychiatric disorder that typically begins in adolescence (Jones, 2013). Despite decades of clinical and preclinical research, the pathogenesis of MDD is still not well understood (Holtzheimer and Nemeroff, 2006). It is now generally accepted that depressive mood tends to follow a seasonal pattern, peaking in summer and winter (Wehr and Rosenthal, 1989). It is likely that vitamin D, through its action on the brain, might account for the link between seasonal photoperiod changes and seasonal mood swings (Berk et al., 2007). Fundamentally, there is abundant evidence for an intimate association between vitamin D deficiency and MDD (Bertone-Johnson et al., 2011; Kjaergaard et al., 2011; Anglin et al., 2013; Ju et al., 2013; Milaneschi et al., 2014; Aghajafari et al., 2018; Wong et al., 2018; Briggs et al., 2019; Zhu et al., 2019). Moreover, longitudinal studies have documented that vitamin D supplementation is beneficial for the treatment of depression (de Koning et al., 2015; Gowda et al., 2015; Casseb et al., 2019; Alghamdi et al., 2020). These findings converge to support the pivotal role of vitamin D in the pathophysiology of MDD.

There is intense research on the potential pathways through which vitamin D exerts its effects on brain development and functioning. One plausible mechanism lies in the fact that vitamin D status affects the synthesis and release of key neurotransmitters in the brain, such as dopamine and serotonin (Eyles et al., 2013; Patrick and Ames, 2014). Acting as a neurosteroid hormone, vitamin D may also play a regulatory role in neurotransmission, neuroprotection, and neuroimmunomodulation (Fernandes de Abreu et al., 2009). In addition, metabolites of vitamin D can cross the blood-brain barrier and bind to vitamin D receptors, which are widely distributed in the brain (Eyles et al., 2005). Given the prior reports that vitamin D is related to both depression and the brain, it is appealing to investigate the neural substrates underlying the association between vitamin D and MDD. Although our earlier preliminary work showed that the relationship between serum concentration of vitamin D (SCVD) and depressive symptoms was mediated by total intracranial volume in MDD patients (Zhu et al., 2019), it offers insufficient material to attempt a more thorough characterization of such neural underpinnings.

Epidemiological studies demonstrate that females are more likely to develop depression than males (Nolen-Hoeksema and Girgus, 1994; Ferrari et al., 2013). While marked gender difference in depression has long been a topic of active investigation, the exact mechanisms responsible for this gender dimorphism remain elusive. Possible explanations include but not limited to gender divergence in the monoamine systems, stress responses, hypothalamus-pituitary-adrenal (HPA) axis function, and the levels of gonadal steroid hormones (e.g., estrogens) (Amin et al., 2005; Solomon and Herman, 2009; Bourke et al., 2012; Fernandez-Guasti et al., 2012; Bangasser and Valentino, 2014; Zagni et al., 2016). In parallel, a large number of studies have reported gender difference in vitamin D status characterized by the fact that SCVD is lower in females than males (Milaneschi et al., 2010; Toffanello et al., 2014b; Song et al., 2016; Yan et al., 2019; Choi et al., 2020; Rhee et al., 2020). Moreover, researchers have demonstrated a gender-dependent relationship between SCVD and depression, with most studies showing a strong and specific SCVD-depression association in females (Milaneschi et al., 2010; Toffanello et al., 2014b; Serati et al., 2016; Amini et al., 2019; Boulkrane et al., 2020). It is well established that vitamin D is related to the production/release of gonadal hormones (Kinuta et al., 2000; Lorenzen et al., 2017), which may offer a mechanistic account for the gender-dependent association between SCVD and MDD.

Previous data have provided solid evidence for a link between vitamin D and cognition, such that vitamin D deficiency is associated with worse cognitive performance in healthy adults (Balion et al., 2012; Annweiler et al., 2013; Mayne and Burne, 2019) and low vitamin D status is causally related to cognitive decline in older adults (Llewellyn et al., 2011; Slinin et al., 2012; Toffanello et al., 2014a; Miller et al., 2015; Pavlovic et al., 2018). Furthermore, a randomized trial revealed that high dose vitamin D supplementation enhanced cognitive ability in healthy adults, especially among those with insufficient vitamin D at baseline (Pettersen, 2017). It is largely known that cognitive deficits are commonly present in MDD patients (Bortolato et al., 2016; Pan et al., 2019). Past research suggests that cognitive dysfunction persists following symptomatic remission (Bortolato et al., 2016) and retention of cognitive impairment may interact with existing emotional and social vulnerability, increasing the risk of recurrent depressive episodes (Knight and Baune, 2018). As such, cognitive symptoms are considered suitable targets and primary outcomes of psychological and pharmacological treatments in MDD (Knight and Baune, 2018). Collectively, it is natural to assume a potential association between vitamin D, cognitive impairment, and depression. Despite recent attempts to clarify this issue (Lerner et al., 2018; Roy, 2021), such association is rather complex and warrants further investigation.

As a non-invasive imaging technique, resting-state functional magnetic resonance imaging (rs-fMRI) has been frequently used to measure spontaneous brain activity based on the blood-oxygen-level-dependent (BOLD) signal (Biswal et al., 1995). The human brain comprises multiple functional networks subserving different functions (Damoiseaux et al., 2006; Power et al., 2011), each network consisting of several brain regions demonstrating similar patterns of BOLD signal change over time whereas different networks showing distinct patterns. Independent component analysis (ICA) is a useful data-driven approach that can be applied to brain rs-fMRI data to extract different functional networks and further examine large-scale inter- and intranetwork functional connectivity (Calhoun et al., 2001a,b; van de Ven et al., 2004; Wang et al., 2020; Cai et al., 2021). By leveraging this approach, investigators have shown functional dysconnectivity of multiple brain networks in MDD (Sacchet et al., 2016; Wu et al., 2017; Albert et al., 2019; Chen et al., 2019; Liu et al., 2019, 2020, 2021; Yu et al., 2019; Jiao et al., 2020).

The objective of the current study was to investigate the relationship between gender, SCVD, clinical manifestations, and functional network connectivity in a large sample of MDD patients and healthy subjects. On the basis of previous literature, we aimed to test three hypotheses: (1) a diagnosis by gender interaction for SCVD, (2) a gender-specific association of SCVD with clinical manifestations and MDD-related functional network connectivity changes, and (3) a mediative role of MDD- and SCVD-related functional network connectivity alterations in accounting for the association between SCVD and clinical manifestations in a gender-specific manner.

## Materials and Methods

### Participants

Major depressive disorder patients were enrolled from Affiliated Psychological Hospital of Anhui Medical University. Healthy controls (HC) were recruited from the local community via poster advertisements. A total of 244 participants with right-handedness were included, consisting of 122 MDD patients and 122 gender- and age-matched HC. Two well-trained clinical psychiatrists confirmed the diagnoses of depression using the MINI-International Neuropsychiatric Interview (M.I.N.I.) in accordance with the International Classification of Diseases (ICD-10) criteria. HC were carefully screened to confirm an absence of any psychiatric illness using the M.I.N.I. The exclusion criteria for all participants were (1) the presence of other psychiatric disorders, e.g., schizophrenia, bipolar disorder, substance-induced mood disorder, anxiety disorders, substance abuse or dependence; (2) a history of severe physical or neurological diseases; (3) a history of head injury leading to a loss of consciousness; (4) contraindications for magnetic resonance imaging (MRI). Additional exclusion criterion for HC was a family history of serious neurological or psychiatric illnesses among their first-degree relatives. It is noteworthy that participants with SCVD values greater than mean + 3 × standard deviation (SD) or smaller than mean − 3 × SD (i.e., outliers, *n* = 3) were excluded. This brought the final sample into 241 participants, comprising 122 MDD patients and 119 HC. The 24-item Hamilton Rating Scale for Depression (HAMD) (Williams, 1988) and the 14-item Hamilton Rating Scale for Anxiety (HAMA) (Thompson, 2015) were utilized to evaluate the severity of depression and anxiety symptoms. All MDD patients were receiving regular antidepressant medications, including selective serotonin reuptake inhibitors (SSRIs), serotonin-norepinephrine reuptake inhibitors (SNRIs), and noradrenergic and specific serotonergic antidepressants (NaSSA). The ethics committee of The First Affiliated Hospital of Anhui Medical University approved this study. After being given a complete description of the study, written informed consent was obtained from all participants.

### Cognitive Assessment

There is convergent evidence that cognitive impairments in prospective memory (PM) and sustained attention appear to be typical characteristics in depression (Han et al., 2012; Rock et al., 2014; Zhou et al., 2017; McFarland and Vasterling, 2018), such that we examined these two cognitive functions here. The cognitive assessment was conducted by a psychiatrist trained in neuropsychological testing. PM is defined as the ability to remember to carry out an intended action after a delay without any explicit instruction (Einstein and McDaniel, 1990; McDaniel and Einstein, 2011). PM is commonly classified into event-based prospective memory (EBPM; i.e., the ability to remember to carry out an intended action at the occurrence of a certain event) and time-based prospective memory (TBPM; i.e., the ability to remember to carry out an intended action at a certain time). Details of the EBPM and TBPM tests can be found in prior literature (Cheng et al., 2013; Yang et al., 2015). For the EBPM test, the experimental stimuli were 30 cards with each containing 12 Chinese words, of which 10 belonged to one major category and the other two to one minor category. Participants were instructed to read out the two words belonging to the minor category on each card. If the selected words belonged to the category of animals (target events), participants were required to tap the table. After the test, participants should write down their telephone number without any hints at the end of the test. There were six target cards (card numbers 5, 10, 15, 20, 24, and 29) in this test. Participants received one point for each correct response to a target card, and they received two points for remembering to write down their phone number. The maximum score was 8 points. For the TBPM test, the experimental stimuli were 100 cards with each containing 12 two-digit numbers. Participants were asked to pick out the smallest and the largest numbers on each card and to tap the table at the target time points (5, 10, and 15 min after the start of the test). The test stopped at the 17-min time point. Participants received two points if they responded within 10 s around the target time, and one point if within 30 s. The maximum score was 6 points.

Sustained attention was measured with a computerized version of the Continuous Performance Task-Identical Pairs (CPT-IP) (Cornblatt et al., 1988). The stimuli were 2−, 3−, or 4-digit numbers in separate conditions, generating separate scores reflecting increasing memory load on digit span. Participants were asked to monitor numbers on a computer screen and respond to any consecutive presentation of identical stimuli by key pressing as quickly as possible. Responses to target trials (pairs that were identical and required a response) and catch trials (pairs that were similar but not identical) were scored as true and false positive responses. The main outcome variable of interest was *d*′ -a well-established discrimination sensitivity index incorporating both true and false positive responses. *d*′ was calculated as *z*(hit rate) − *z*(false alarm rate) [in Matlab, norminv(hit rate) − norminv(false alarm rate)]. CPT-IP-2, −3, and −4 represented *d*′ values corresponding to the number of digits, with higher scores reflecting better sustained attention performance.

### Serum Concentration of Vitamin D Measurement

After an overnight fasting period, we collected peripheral venous blood samples (2 ml) from all participants in the morning of MRI scanning. Samples were sent to the Department of Clinical Laboratory, Affiliated Psychological Hospital of Anhui Medical University immediately for centrifugation to separate serum. Serum vitamin D [25(OH)D] was measured using a chemiluminescence immunoassay (CLIA) technique in a fully automated Maglumi 1000 analyzer (SNIBE Co., Ltd., Shenzhen, China). Internal quality control provided by the manufacturer was employed to assure quality. SCVD was stratified as sufficiency: 30–100 ng/ml (75–250 nmol/L), insufficiency: 20–30 ng/ml (50–75 nmol/L), and deficiency: <20 ng/ml (50 nmol/L) (Ringe and Kipshoven, 2012).

### Image Acquisition

Magnetic resonance imaging data were acquired using a 3.0-Tesla MR system (Discovery MR750w, General Electric, Milwaukee, WI, United States) with a 24-channel head coil. During scanning, tight but comfortable foam and earplugs were used to minimize head movement and scanner noise. All participants were instructed to relax, keep their eyes closed but not fall asleep, think of nothing in particular, and move as little as possible. All participants underwent a high-resolution three-dimensional T1-weighted brain volume (BRAVO) sequence with the following parameters: repetition time (TR) = 8.5 ms; echo time (TE) = 3.2 ms; inversion time (TI) = 450 ms; flip angle (FA) = 12°; field of view (FOV) = 256 mm × 256 mm; matrix size = 256 × 256; slice thickness = 1 mm, no gap; voxel size = 1mm × 1mm × 1mm; 188 sagittal slices; and acquisition time = 296 s. Resting-state BOLD fMRI data were acquired using a gradient-echo single-shot echo planar imaging (GRE-SS-EPI) sequence with the following parameters: TR = 2,000 ms; TE = 30 ms; FA = 90°; FOV = 220 mm × 220 mm; matrix size = 64 × 64; slice thickness = 3 mm, slice gap = 1 mm; 35 interleaved axial slices; 185 volumes; and acquisition time = 370 s. Routine T2-weighted images were also collected to exclude any organic brain abnormality. All images were visually inspected to ensure that only images without visible artifacts (e.g., ghosting artifacts arising from subject movement and pulsating large arteries, metal artifacts, susceptibility artifacts, and blooming artifacts) were included in subsequent analyses. None of the participants were excluded for visually inspected imaging artifacts.

### fMRI Data Preprocessing

Resting-state fMRI data were preprocessed using Statistical Parametric Mapping software (SPM12^1^) and Data Processing and Analysis for Brain Imaging (DPABI^2^) (Yan et al., 2016). The first 10 volumes for each participant were discarded, and the remaining volumes were corrected for the acquisition time delay between slices. Realignment was then performed to correct the motion between time points. Head motion parameters were computed by estimating the translation in each direction and the angular rotation on each axis for each volume. All participants’ BOLD data were within the defined motion thresholds (i.e., maximum translational or rotational motion parameters less than 2 mm or 2°). We also calculated FD, which indexes the volume-to-volume changes in head position. In the normalization step, individual structural images were firstly co-registered with the mean functional image; the transformed structural images were then segmented and normalized to the Montreal Neurological Institute (MNI) space using a high-level non-linear warping algorithm, i.e., the diffeomorphic anatomical registration through exponentiated Lie algebra (DARTEL) technique (Ashburner, 2007). Finally, each functional volume was spatially normalized to the MNI space using the deformation parameters estimated during the above step and resampled into a 3-mm isotropic voxel. After spatial normalization, all data sets were smoothed with a 6 mm full-width at half-maximum (FWHM) Gaussian kernel.

### Independent Component Analysis

Independent component analysis was performed for the preprocessed fMRI data with the GIFT toolbox^3^ and the number of independent components (*N* = 21) was estimated automatically by the software using the minimum description length criteria. Spatial ICA decomposes the participant data into linear mixtures of spatially independent components that exhibit a unique time course profile. This was realized by using two data reduction steps. First, principal component analysis was adopted to reduce the subject-specific data into 32 principle components. Next, reduced data of all participants were concatenated across time and decomposed into 21 independent components using the infomax algorithm. To ensure estimation stability, the infomax algorithm was repeated 20 times in ICASSO^4^, and the most central run was selected and analyzed further. Finally, participant specific spatial maps and time courses were obtained using the GICA back reconstruction approach.

**FIGURE 1:**
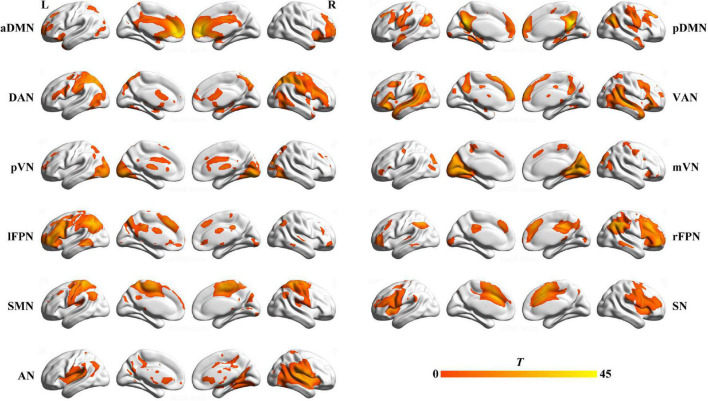
Spatial maps of 11 selected functional networks. aDMN, anterior default mode network; pDMN, posterior default mode network; DAN, dorsal attention network; VAN, ventral attention network; pVN, posterior visual network; mVN, medial visual network; lFPN, left frontoparietal network; rFPN, right frontoparietal network; SMN, sensorimotor network; SN, salience network; AN, auditory network; L, left; R, right.

We identified several independent components as functional networks. These independent components had peak activations in gray matter, showed low spatial overlap with known vascular, ventricular, motion, and susceptibility artifacts, and exhibited primarily low frequency power. This selection procedure yielded 11 functional networks out of the 21 independent components obtained (Figure 1): anterior and posterior default mode networks (aDMN and pDMN), dorsal and ventral attention networks (DAN and VAN), posterior and medial visual networks (pVN and mVN), left and right frontoparietal networks (lFPN and rFPN), sensorimotor network (SMN), salience network (SN), and auditory network (AN).

Before internetwork functional connectivity calculation, the following additional postprocessing steps were carried out on the time courses of selected functional networks: (1) detrending linear, quadratic, and cubic trends; (2) despiking detected outliers; and (3) low-pass filtering with a cut-off frequency of 0.15 Hz. Then, internetwork functional connectivity was estimated as the Pearson correlation coefficients between pairs of time courses of the functional networks, resulting in a symmetric 11 × 11 correlation matrix per participant. Finally, correlations were transformed to *z*-scores using Fisher’s transformation to improve the normality. Intranetwork connectivity was examined via the spatial maps, indexing the contribution of the time course to each voxel comprising a given independent component.

### Statistical Analysis

Demographic and clinical data were analyzed using the SPSS 23.0 software package (SPSS, Chicago, IL, United States). Two-sample *t*-tests were utilized to compare MDD patients and HC in age, educational level, body mass index (BMI), FD, HAMD, HAMA, EBPM, TBPM, and CPT-IP. Pearson Chi-square test was adopted to examine group difference in gender. A threshold of *P* < 0.05 was considered statistically significant (two-sided).

Since our principal focus was set on the group × gender interaction effect on SCVD, a general linear model was utilized to examine this effect with age and education as nuisance variables. In case of significant interaction, we performed *post hoc* pairwise comparisons in SCVD between patients and HC in females and males, respectively. For a gender exhibiting significant group differences, subsequent analyses examining the relationships of SCVD with clinical and neuroimaging data were conducted in this gender. With respect to clinical data, we examined their associations with SCVD using partial correlation analyses controlling for age and education. Multiple comparisons were corrected using the false discovery rate (FDR) method with a corrected significance threshold of *P* < 0.05. In terms of neuroimaging data, inter- and intranetwork functional connectivity were analyzed, respectively. Briefly, differences in internetwork functional connectivity between patients and HC were tested using a general linear model with age, education and FD as nuisance variables. For intranetwork functional connectivity, all participants’ spatial maps for each functional network were initially entered into a random-effect one-sample *t*-test. Brain regions were considered to be within each network if they met a height threshold of *P* < 0.05 corrected for multiple comparisons using a family-wise error (FWE) method and an extent threshold of 100 voxels. Within each network, a voxel-wise two-sample *t*-test was then used to examine intranetwork connectivity differences between patients and HC controlling for age, education and FD. Multiple comparisons were corrected using the cluster-level FWE method, resulting in a cluster defining threshold of *P* = 0.001 and a corrected cluster significance of *P* < 0.05. After group comparisons, inter- and intranetwork functional connectivity with significant group differences were extracted for subsequent correlation analyses with SCVD. Finally, we further tested the associations of the SCVD-sensitive functional network connectivity with clinical variables.

To examine the potential relationship among SCVD, functional network connectivity and clinical variables, we tested the SCVD-functional connectivity-clinical variables mediation model where functional connectivity mediated the relation between SCVD and clinical variables. The PROCESS macro^5^ available for SPSS (Hayes, 2009, 2014) was used to conduct the mediation analyses. Only variables that showed a significant correlation with others were considered independent, dependent or mediating variables in the mediation analyses. Age, education and FD were included as nuisance variables. Based on 5,000 bootstrap realizations, the significance of mediation effects was determined by the bootstrap 95% confidence interval (CI) in the way a significant indirect effect is indicated when the bootstrap 95% CI does not include zero.

### Sensitivity and Specificity Analyses

Considering a significant effect of BMI on SCVD (Wortsman et al., 2000), we repeated the above-mentioned analyses after additionally adjustment for BMI. To test the specificity of our results, we also conducted the same analyses in the gender demonstrating no group effect on SCVD.

## Results

### Demographic and Clinical Characteristics

In agreement with our hypothesis, a significant interaction effect (*F* = 6.050, *P* = 0.015) of group × gender on SCVD was observed (Supplementary Figure 1). *Post hoc* analyses demonstrated that MDD patients presented with a significant SCVD reduction compared with HC in females (*P* < 0.001) but not males (*P* > 0.05), indicative of a gender-specific effect. As such, we focused our subsequent analyses on females. Demographic and clinical characteristics of the females are shown in Table 1. Briefly, there were no significant differences in age (two-sample *t*-test, *t* = 0.061, *P* = 0.952), BMI (*t* = −1.076, *P* = 0.284), and FD (*t* = −0.003, *P* = 0.998) between female patients and controls. Nevertheless, female MDD patients showed lower SCVD (*t* = −5.940, *P* < 0.001), educational level (*t* = −4.100, *P* < 0.001), EBPM (*t* = −7.638, *P* < 0.001), TBPM (*t* = −11.482, *P* < 0.001), CPT-IP-2 (*t* = −6.559, *P* < 0.001), CPT-IP-3 (*t* = −4.954, *P* < 0.001), and CPT-IP-4 (*t* = −3.273, *P* = 0.001), and higher HAMD (*t* = 22.244, *P* < 0.001) and HAMA (*t* = 21.287, *P* < 0.001) than female HC. Among 82 female patients, 11 (13.41%) were classified as vitamin D insufficiency and 70 (85.37%) as deficiency. Additionally, demographic and clinical characteristics of all participants are listed in Supplementary Table 1

**TABLE 1:** Demographic and clinical characteristics of the females.

Characteristics	MDD (*n* = 82)	HC (*n* = 82)	Statistics	*P*-value
Age (years)	44.35 ± 10.28 (21–62)	44.24 ± 12.75 (21–62)	*t* = 0.061	0.952
Education (years)	8.45 ± 3.76 (0–16)	11.15 ± 4.63 (3–20)	*t* = −4.100	<0.001
BMI (kg/m^2^)	22.39 ± 3.66 (13.93–32.44)	22.93 ± 2.67 (15.98–31.22)	*t* = −1.076	0.284
HAMD	30.06 ± 11.25 (1–52)	1.49 ± 2.97 (0–19)	*t* = 22.244	<0.001
HAMA	20.68 ± 7.40 (2–35)	1.63 ± 3.31 (0–21)	*t* = 21.287	<0.001
EBPM	2.28 ± 2.55 (0–8)	5.49 ± 2.82 (0–8)	*t* = −7.638	<0.001
TBPM	1.89 ± 2.27 (0–6)	5.30 ± 1.45 (0–6)	*t* = −11.482	<0.001
CPT-IP-2	2.07 ± 1.09 (−0.12–4.24)	3.11 ± 0.93 (1.11–4.24)	*t* = −6.559	<0.001
CPT-IP-3	1.50 ± 0.97 (−0.26–4.24)	2.29 ± 1.08 (0.28–4.24)	*t* = −4.954	<0.001
CPT-IP-4	0.85 ± 0.69 (−0.41–3.12)	1.24 ± 0.83 (−0.44–3.34)	*t* = −3.273	0.001
SCVD (nmol/L)	38.87 ± 12.05 (15.95–78.36)	50.91 ± 15.83 (21.50–86.25)	*t* = −5.940	<0.001
FD (mm)	0.13 ± 0.09 (0.05–0.60)	0.13 ± 0.07 (0.06–0.40)	*t* = −0.003	0.998
Illness duration (months)	62.74 ± 71.44 (0.30–306)	–	–	–
Onset age (years)	38.95 ± 10.46 (19–55)	–	–	–
Episode number	2.52 ± 2.40 (1–21)	–	–	–
Antidepressant medications				
SSRIs	58	–	–	–
SNRIs	19	–	–	–
NaSSA	5	–	–	–

*Data are expressed as means ± standard deviations. Numbers in parentheses are the range. MDD, major depressive disorder; HC, healthy controls; BMI, body mass index; HAMD, Hamilton Rating Scale for Depression; HAMA, Hamilton Rating Scale for Anxiety; EBPM, event-based prospective memory; TBPM, time-based prospective memory; CPT-IP, Continuous Performance Task-Identical Pairs; SCVD, serum concentration of vitamin D; FD, frame-wise displacement; SSRIs, selective serotonin reuptake inhibitors; SNRIs, serotonin norepinephrine reuptake inhibitors; NaSSA, noradrenergic and specific serotonergic antidepressant.*

### Associations Between Serum Concentration of Vitamin D and Clinical Variables in Females

There were significant positive correlations of SCVD with EBPM (partial correlation coefficient [*pr*] = 0.267, *P* < 0.001), TBPM (*pr* = 0.355, *P* < 0.001), and CPT-IP-2 (*pr* = 0.215, *P* = 0.006) in females (*P* < 0.05, FDR corrected). Correlations between SCVD and clinical symptoms (HAMD and HAMA) were not observed in female MDD patients (Supplementary Table 2).

### Group Differences of Functional Network Connectivity in Females

In comparison with female HC, female MDD patients manifested reduced functional connectivity between AN and DAN (*F* = 3.987, *P* = 0.048) and between pVN and lFPN (*F* = 7.007, *P* = 0.009) as well as increased connectivity between AN and aDMN (*F* = 4.068, *P* = 0.045), between VAN and SMN (*F* = 6.230, *P* = 0.014) and between SMN and SN (*F* = 7.709, *P* = 0.006).

Voxel-wise intranetwork functional connectivity analyses revealed that female MDD patients exhibited increased connectivity in the bilateral lateral parietal cortex (LPC) of DAN, the right anterior angular gyrus (aANG) and posterior angular gyrus (pANG) of pDMN, the right inferior parietal gyrus (IPG) of rFPN, the left middle cingulate cortex (MCC) of SMN, and the right calcarine sulcus (CAL) of mVN relative to female HC (*P* < 0.05, cluster-level FWE corrected) (Table 2 and Supplementary Figure 2).

**TABLE 2:** Brain regions showing increased intranetwork functional connectivity in female MDD patients relative to female HC.

Regions	Cluster size (voxels)	Peak *t-*values	Coordinates in MNI (*x-*, *y*-, *z-*)
DAN			
Left lateral parietal cortex	108	4.688	−27, −69, 39
Right lateral parietal cortex	437	6.650	33, −66, 42
pDMN			
Right anterior angular gyrus	60	5.176	54, −60, 30
Right posterior angular gyrus	29	4.615	36, −63, 45
rFPN			
Right inferior parietal gyrus	53	4.699	39, −45, 51
SMN			
Left middle cingulate cortex	29	4.567	−12, −21, 42
mVN			
Right calcarine sulcus	28	3.757	15, −81, 12

*DAN, dorsal attention network; pDMN, posterior default mode network; rFPN, right frontoparietal network; SMN, sensorimotor network; mVN, medial visual network; MDD, major depressive disorder; HC, healthy controls; MNI, Montreal Neurological Institute.*

### Associations Between Serum Concentration of Vitamin D and Functional Network Connectivity in Females

**FIGURE 2:**
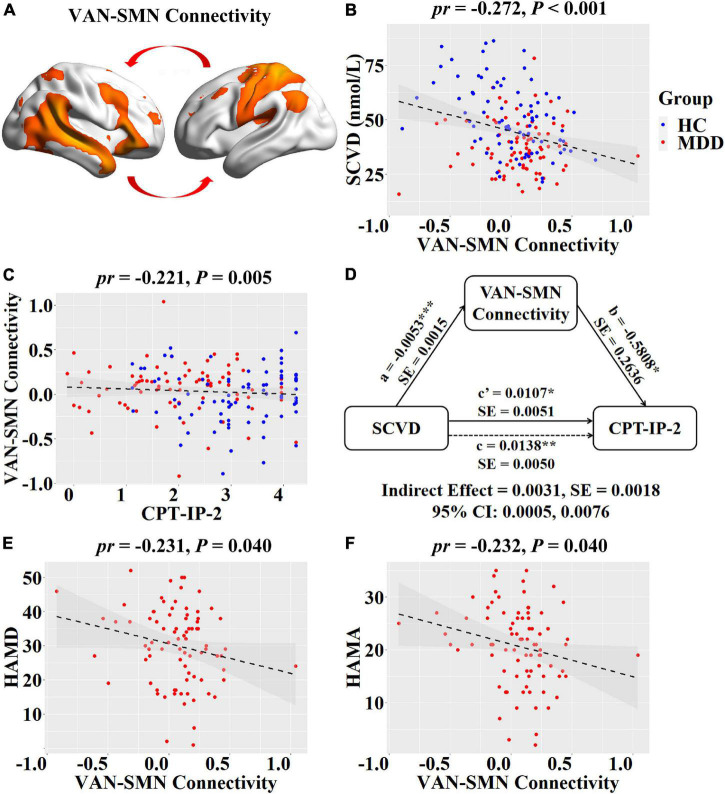
Associations between SCVD, internetwork functional connectivity, and clinical variables in females. **(A)** Female patients and controls differed in functional connectivity between VAN and SMN. **(B)** A scatter plot shows correlation between SCVD and VAN-SMN connectivity in females. **(C)** A scatter plot shows correlation between VAN-SMN connectivity and CPT-IP-2 in females. **(D)** Graphical representation of the mediation analysis between SCVD and CPT-IP-2 in females with VAN-SMN connectivity as mediator: estimates of the mediated (*a* × *b*), direct (*c*′), and total (c) effects. **(E,F)** Scatter plots show correlations of VAN-SMN connectivity with HAMD and HAMA scores. *P < 0.05, **P < 0.01, ***P < 0.001. SCVD, serum concentration of vitamin D; VAN, ventral attention network; SMN, sensorimotor network; CPT-IP, Continuous Performance Task-Identical Pairs; HAMD, Hamilton Rating Scale for Depression; HAMA, Hamilton Rating Scale for Anxiety; HC, healthy controls; MDD, major depressive disorder; *pr*, partial correlation coefficient; SE, standard error; CI, confidence interval.

**FIGURE 3:**
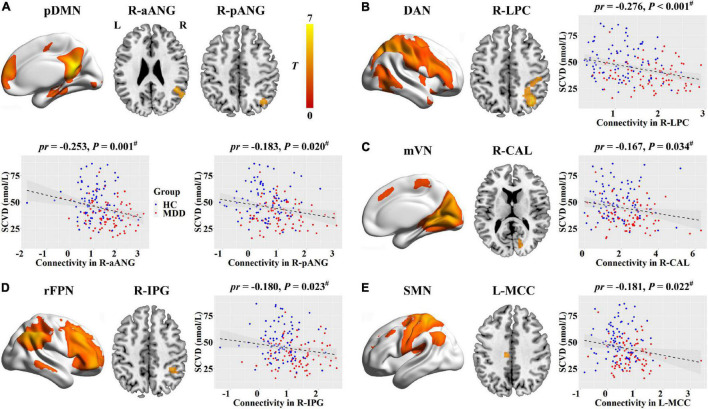
Associations between SCVD and intranetwork functional connectivity in females. **(A–E)** SCVD was correlated with intranetwork connectivity in brain regions showing group differences; scatter plots show the correlations between SCVD and intranetwork connectivity. The warm color denotes increased intranetwork connectivity in MDD patients. ^#^*P* < 0.05, false discovery rate correction for multiple comparisons. SCVD, serum concentration of vitamin D; pDMN, posterior default mode network; aANG, anterior angular gyrus; pANG, posterior angular gyrus; DAN, dorsal attention network; LPC, lateral parietal cortex; mVN, medial visual network; CAL, calcarine sulcus; rFPN, right frontoparietal network; IPG, inferior parietal gyrus; SMN, sensorimotor network; MCC, middle cingulate cortex; HC, healthy controls; MDD, major depressive disorder; L, left; R, right.

For internetwork functional connectivity, SCVD was negatively correlated with VAN-SMN connectivity (*pr* = −0.272, *P* < 0.001, Figures 2A,B) in females. For intranetwork functional connectivity, SCVD was negatively correlated with connectivity in the right aANG (*pr* = −0.253, *P* = 0.001, Figure 3A) and pANG (*pr* = −0.183, *P* = 0.020, Figure 3A) of pDMN, the right LPC (*pr* = −0.276, *P* < 0.001, Figure 3B) of DAN, the right CAL (*pr* = −0.167, *P* = 0.034, Figure 3C) of mVN, the right IPG (*pr* = −0.180, *P* = 0.023, Figure 3D) of rFPN, and the left MCC (*pr* = −0.181, *P* = 0.022, Figure 3E) of SMN in females (*P* < 0.05, FDR corrected).

### Associations Between Serum Concentration of Vitamin D, Functional Network Connectivity and Clinical Variables in Females

For internetwork functional connectivity, VAN-SMN connectivity was negatively correlated with CPT-IP-2 (*pr* = −0.221, *P* = 0.005, Figure 2C) in females. In the mediation analysis model, all paths were reported as unstandardized ordinary least squares regression coefficients, namely, total effect of *X* on *Y* (*c*) = indirect effect of *X* on *Y* through *M* (*a* × *b*) + direct effect of *X* on *Y* (*c*′). We found that the relationship between SCVD and CPT-IP-2 was significantly mediated by VAN-SMN connectivity (indirect effect = 0.0031, *SE* = 0.0018, 95% CI: 0.0005, 0.0076, Figure 2D) in females. Moreover, we observed significant negative correlations of VAN-SMN connectivity with HAMD (*pr* = −0.231, *P* = 0.040, Figure 2E) and HAMA (*pr* = −0.232, *P* = 0.040, Figure 2F) in female MDD patients.

**FIGURE 4:**
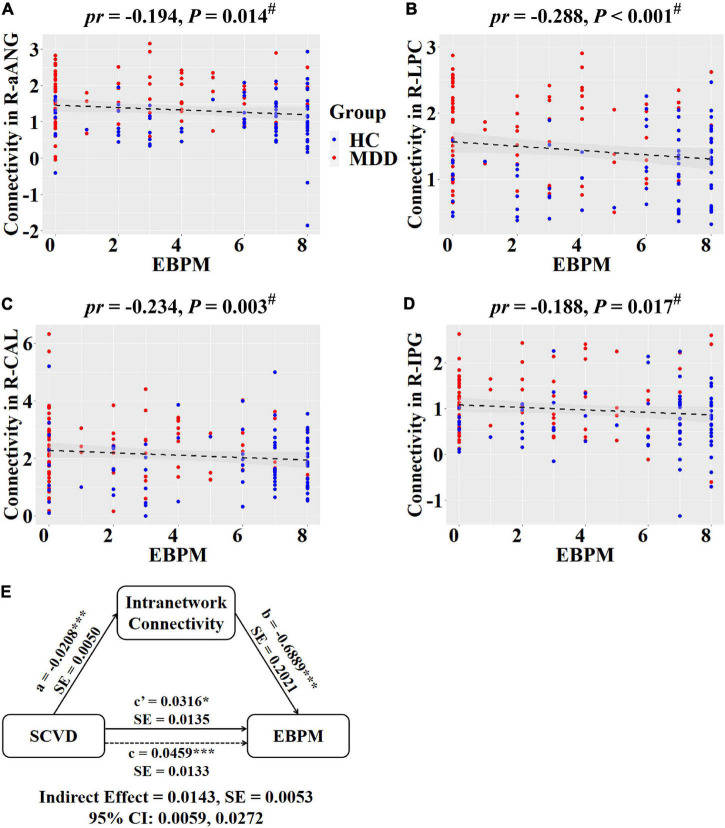
Associations between SCVD, intranetwork functional connectivity, and EBPM in females. **(A–D)** Scatter plots show correlations between SCVD-related intranetwork connectivity and EBPM. **(E)** Graphical representation of the mediation analysis between SCVD and EBPM with first intranetwork functional connectivity component as the mediator: estimates of the mediated (*a* × *b*), direct (*c*′), and total (c) effects. ^#^*P* < 0.05, false discovery rate correction for multiple comparisons. *P < 0.05, ***P < 0.001. SCVD, serum concentration of vitamin D; EBPM, event-based prospective memory; aANG, anterior angular gyrus; LPC, lateral parietal cortex; CAL, calcarine sulcus; IPG, inferior parietal gyrus; HC, healthy controls; MDD, major depressive disorder; R, right; *pr*, partial correlation coefficient; SE, standard error; CI, confidence interval.

**FIGURE 5:**
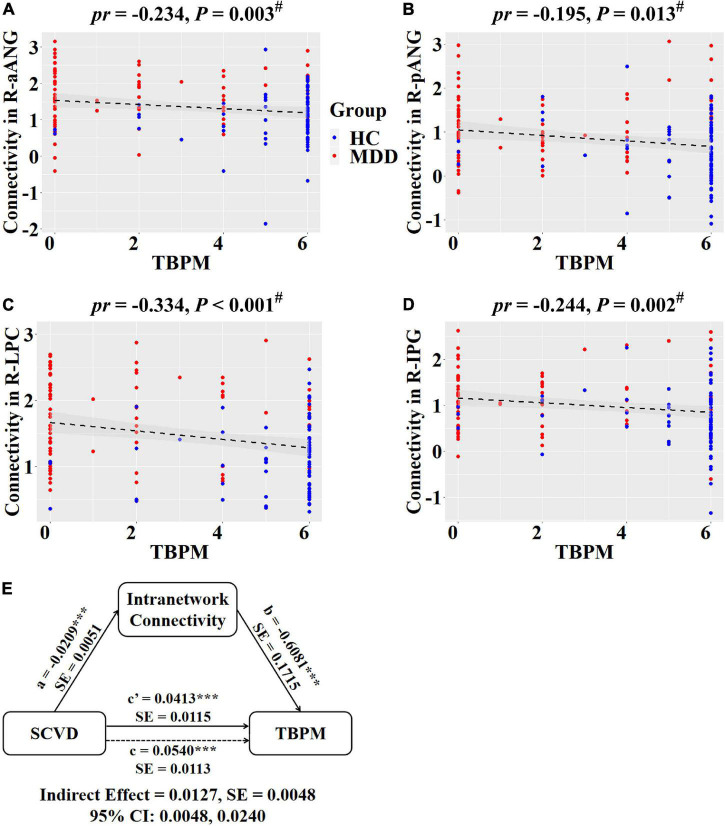
Associations between SCVD, intranetwork functional connectivity, and TBPM in females. **(A–D)** Scatter plots show correlations between SCVD-related intranetwork connectivity and TBPM. **(E)** Graphical representation of the mediation analysis between SCVD and TBPM with first intranetwork functional connectivity component as the mediator: estimates of the mediated (*a* × *b*), direct (*c*′), and total (*c*) effects. ^#^*P* < 0.05, false discovery rate correction for multiple comparisons. ***P < 0.001. SCVD, serum concentration of vitamin D; TBPM, time-based prospective memory; aANG, anterior angular gyrus; pANG, posterior angular gyrus; LPC, lateral parietal cortex; IPG, inferior parietal gyrus; HC, healthy controls; MDD, major depressive disorder; R, right; *pr*, partial correlation coefficient; SE, standard error; CI, confidence interval.

**FIGURE 6:**
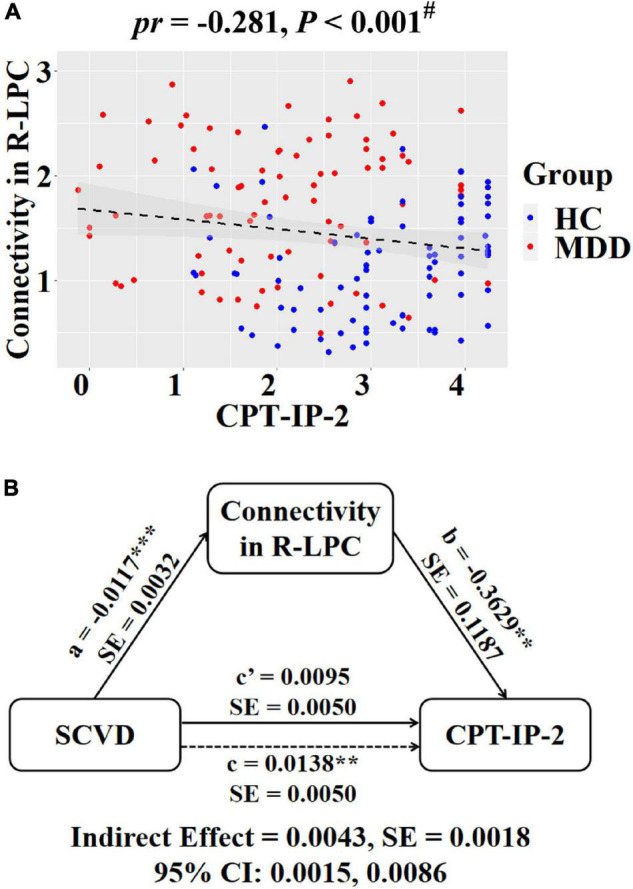
Associations between SCVD, intranetwork functional connectivity, and CPT-IP-2 in females. **(A)** A scatter plot shows correlation between intranetwork connectivity in the right LPC and CPT-IP-2. **(B)** Graphical representation of the mediation analysis between SCVD and CPT-IP-2 with intranetwork connectivity in the right LPC as the mediator: estimates of the mediated (*a* × *b*), direct (*c*′), and total (*c*) effects. ^#^*P* < 0.05, false discovery rate correction for multiple comparisons. **P < 0.01, ***P < 0.001. SCVD, serum concentration of vitamin D; CPT-IP, Continuous Performance Task-Identical Pairs; LPC, lateral parietal cortex; HC, healthy controls; MDD, major depressive disorder; R, right; *pr*, partial correlation coefficient; SE, standard error; CI, confidence interval.

Regarding intranetwork functional connectivity, EBPM was negatively correlated with connectivity in the right aANG (*pr* = −0.194, *P* = 0.014, Figure 4A), right LPC (*pr* = −0.288, *P* < 0.001, Figure 4B), right CAL (*pr* = −0.234, *P* = 0.003, Figure 4C), and right IPG (*pr* = −0.188, *P* = 0.017, Figure 4D) in females (*P* < 0.05, FDR corrected). To summarize individual differences in intranetwork functional connectivity, a principal component analysis (PCA) was performed to identify latent components underlying the EBPM-related intranetwork functional connectivity. Based on the Kaiser-Guttman criterion, components with an eigenvalue (EV) < 1.5 were removed. Consequently, only the first intranetwork connectivity component that accounted for 50.73% of the variance was retained and extracted for subsequent mediation analysis. The relationship between SCVD and EBPM was significantly mediated by the first intranetwork connectivity component (indirect effect = 0.0143, *SE* = 0.0053, 95% CI: 0.0059, 0.0272, Figure 4E) in females. In addition, TBPM was negatively correlated with connectivity in the right aANG (*pr* = −0.234, *P* = 0.003, Figure 5A), right pANG (*pr* = −0.195, *P* = 0.013, Figure 5B), right LPC (*pr* = −0.334, *P* < 0.001, Figure 5C), and right IPG (*pr* = −0.244, *P* = 0.002, Figure 5D) in females (*P* < 0.05, FDR corrected). PCA revealed that the first intranetwork connectivity component accounted for 51.78% of the variance. The relationship between SCVD and TBPM was significantly mediated by the first intranetwork connectivity component (indirect effect = 0.0127, *SE* = 0.0048, 95% CI: 0.0048, 0.0240, Figure 5E) in females. Finally, CPT-IP-2 was negatively correlated with connectivity in the right LPC (*pr* = −0.281, *P* < 0.001, Figure 6A) in females (*P* < 0.05, FDR corrected). Moreover, connectivity in the right LPC significantly mediated the relationship between SCVD and CPT-IP-2 (indirect effect = 0.0043, *SE* = 0.0018, 95% CI: 0.0015, 0.0086, Figure 6B) in females.

No significant correlations were found between functional network connectivity and other clinical variables (Supplementary Table 3).

### Sensitivity and Specificity Analyses

After additionally controlling for BMI, the interaction effect of group × gender on SCVD remained significant (*F* = 6.066, *P* = 0.015) and the correlations of SCVD with clinical variables and functional network connectivity were unchanged (Supplementary Tables 4, 5), suggesting no influence of BMI on our results. In males, no significant correlations were found between SCVD and clinical variables (Supplementary Table 6). The correlations between SCVD and functional network connectivity were non-significant or showed opposite directions in males (Supplementary Table 7). In addition, most of the significant correlations between functional network connectivity and clinical variables in females were absent in males (Supplementary Table 8).

## Discussion

Our data represent the first report to date exploring the relationship between gender, SCVD, clinical manifestations, and functional network connectivity in a large sample of MDD patients and HC. There were four main findings observed in the present study. First, there was a significant group-by-gender interaction effect on SCVD in the way MDD patients exhibited lower SCVD relative to HC in females rather than males. Second, we identified a female-specific association between lower SCVD and poorer cognitive performance (i.e., prospective memory and sustained attention). Third, MDD-related functional network connectivity changes were correlated with SCVD in females as well as depression and anxiety symptoms in female patients with MDD. Finally, MDD- and SCVD-related functional network connectivity alterations mediated the associations between SCVD and cognition in females.

A large number of cross-sectional and longitudinal studies have established an intimate link between low vitamin D and depression (Milaneschi et al., 2010, 2014; Anglin et al., 2013; Ju et al., 2013; Aghajafari et al., 2018; Wong et al., 2018; Briggs et al., 2019), indicating the critical role of low vitamin D in the pathophysiology of MDD. Furthermore, vitamin D supplementation has been shown to hold promise as an effective antidepressant intervention approach (de Koning et al., 2015; Gowda et al., 2015; Casseb et al., 2019; Alghamdi et al., 2020). Overall, these previous findings are coincident with our current observation that MDD patients had significantly lower SCVD (vitamin D insufficiency or deficiency) relative to HC in females.

This study revealed that vitamin D insufficiency and deficiency was more common in females than males, which is in line with many previous studies (Milaneschi et al., 2010; Toffanello et al., 2014b; Song et al., 2016; Yan et al., 2019; Choi et al., 2020; Rhee et al., 2020). Several factors might explain this gender difference, such as insufficient sunlight exposure, higher BMI, more fat tissue, and more sedentary life in females relative to males. Interestingly, females are more likely to suffer from depression than males (Nolen-Hoeksema and Girgus, 1994; Ferrari et al., 2013), with the high-risk periods for developing depression including adolescence, pregnancy, parturition, and perimenopause (Kessler, 2003; Woods et al., 2008; Amini et al., 2019). Crucially, these periods are typically characterized by a profound hormonal shift, which indicates the importance of gonadal hormones in the heightened predominance of depression among females. Here, we found that MDD patients presented a lower SCVD than HC in females but not males, suggesting a female-specific involvement of vitamin D in the pathogenesis of depression. Combined, these findings might have led to some speculation that abnormal changes of gonadal hormones in females, arising from low vitamin D status, may lead to the development of depression given that vitamin D is related to the production/release of gonadal hormones (Kinuta et al., 2000; Lorenzen et al., 2017). In support of this view, previous research has provided evidence that the gender difference in prevalence of depression is less apparent in later middle age, particularly during menopause when gonadal hormonal flux stabilizes (Bebbington et al., 1998). Besides, it is evident that hormone replacement therapy during the perimenopausal period can be effective in preventing postmenopausal depression (Gordon and Girdler, 2014).

Cognitive dysfunction represents a characteristic feature of MDD (Bortolato et al., 2016; Pan et al., 2019), which is closely linked to depression severity (McDermott and Ebmeier, 2009) and persists following treatment of affective symptoms as well as increases the risk of recurrent depressive episodes (Knight and Baune, 2018), highlighting the need to treat cognitive impairment separately from mood symptoms. In agreement with previous findings, we found that sustained attention and prospective memory were worse in female MDD patients than female HC. Moreover, the worse cognitive performance was related to lower SCVD, extending prior reports of a potentially causal relationship between low vitamin D and cognitive dysfunction in healthy populations (Llewellyn et al., 2011; Balion et al., 2012; Slinin et al., 2012; Annweiler et al., 2013; Toffanello et al., 2014a; Miller et al., 2015; Pettersen, 2017; Pavlovic et al., 2018; Mayne and Burne, 2019) by unraveling such vitamin D-cognition association in a psychiatric population. The present observation also has clinical implications for the development of a precision medicine approach in antidepressant treatment, i.e., assignment of female patients with comorbid MDD and cognitive deficits to adjuvant vitamin D supplementation therapy.

Major depressive disorder patients have been extensively shown to exhibit resting-state functional connectivity abnormalities involving a wide range of functional networks (Sacchet et al., 2016; Wu et al., 2017; Albert et al., 2019; Chen et al., 2019; Liu et al., 2019, 2020, 2021; Yu et al., 2019; Jiao et al., 2020). In the current study, we identified a complex pattern of decreased (AN-DAN and pVN-lFPN connectivity) and increased (AN-aDMN, VAN-SMN, and SMN-SN connectivity) internetwork functional connectivity as well as increased intranetwork connectivity (DAN, pDMN, rFPN, SMN, and mVN) in female MDD patients. Although some of these functional network connectivity alterations are coherent with prior reports, some are at odds with previous findings of decreased intranetwork functional connectivity in particular. The commonalities and differences with earlier research may be due to the fact that we focused our analysis on females and thus identified functional network connectivity alterations specific to female patients with MDD, whereas earlier research has examined functional network connectivity changes in mixed patients.

Vitamin D plays an important role in neuronal development and brain function as well as the synthesis, release, and regulation of neurotransmitters (Fernandes de Abreu et al., 2009; Eyles et al., 2013; Patrick and Ames, 2014, 2015; Kesby et al., 2017). Vitamin D exerts its effects on the brain via binding to vitamin D receptors (Ryan et al., 2015), which are broadly distributed in the prefrontal cortex, cingulate cortex, and limbic system (Eyles et al., 2005). In recent years, the relationship between vitamin D and the brain has attracted much interest from neuroimaging investigators. For instance, Annweiler et al. (2014) found that vitamin D depletion was associated with smaller brain volume and larger lateral cerebral ventricles. Another structural MRI study reported a negative correlation between vitamin D and intracranial volume as well as total cortical gray and cerebral white matter volumes in healthy young women (Plozer et al., 2015). In older adults, lower vitamin D has been found to relate to lower gray matter volume of the hippocampus (Karakis et al., 2016) and calcarine sulcus (Ali et al., 2020) as well as thinner cingulate cortex (Foucault et al., 2019). Of note, our earlier work identified an association between lower SCVD and more severe depressive symptoms in MDD patients, which was mediated by total intracranial volume (Zhu et al., 2019). Nevertheless, these prior studies place less emphasis on brain functional measures that may represent a more sensitive and specific index of state-related changes and instead focus on structural measures that are more stable and thus may reflect more enduring signatures. By means of functional network connectivity analysis, we observed that lower SCVD was associated with higher VAN-SMN functional connectivity and higher intranetwork connectivity of pDMN, DAN, mVN, rFPN, and SMN that characterized MDD in females. Moreover, higher VAN-SMN functional connectivity was associated with depression and anxiety symptoms in female patients, which is consistent with previous research (Yu et al., 2019). Our data support the concept that brain functional measures are able to more sensitively detect small effects and more closely track symptom expression.

Critically, we found that the MDD- and SCVD-related functional network connectivity changes were related to sustained attention and prospective memory in females. Furthermore, the functional network connectivity alterations mediated the relationship between SCVD and cognitive performance. These findings endorse the notion that complex cognitive functions are supposed to rely on a variety of neural processes arising from integration and segregation of different functional networks. Specifically, sustained attention was related to VAN-SMN functional connectivity and intranetwork connectivity of DAN. DAN is involved in goal-directed (top-down) control of attention and VAN, coupled with sensory systems, is implicated in stimulus-driven control of attention (Corbetta and Shulman, 2002; Vossel et al., 2014). Our results corroborate the importance of both attention networks in attention-demanding cognitive tasks. In addition, EBPM was related to intranetwork connectivity of pDMN, DAN, rFPN and mVN, and TBPM was linked to intranetwork connectivity of pDMN, DAN and rFPN. DMN can be typically divided into anterior and posterior sub-networks (Andrews-Hanna et al., 2010), with pDMN predominantly engaged in memory processing (Andrews-Hanna et al., 2014; Raichle, 2015). FPN appears to be most active during cognitive tasks involving working memory (Mulders et al., 2015). Notably, the prominent involvement of mVN in EBPM but not TBPM may be attributable to the fact that detecting and processing of visual stimuli are a prerequisite for EBPM tasks. Altogether, our data might help elucidate the potential neural mechanisms by which lower SCVD contributes to cognitive dysfunction in female MDD patients.

There are several limitations that should be mentioned. First, our results might be influenced by the confounds of antidepressant treatment and illness chronicity. Future studies in a sample of first-episode, medication-naive patients with MDD are required to validate our preliminary findings. Second, despite several independent components identified as meaningful functional networks according to a strict selection procedure, there are possible biases, e.g., some non-typical but physiologically important functional networks might have been ignored. Third, MDD patients and HC differed significantly in educational level. While education was included as a covariate of no interest in our analyses, its potential residual effects cannot be ruled out completely. Fourth, it should be noted that patients with anxiety disorders were excluded. Since anxiety is frequently comorbid with MDD, this reduces the generalizability of the findings to the general population with MDD. Fifth, this study is cross-sectional and therefore cannot discern between cause and effect. Longitudinal studies are necessary for determining causality between determinant and outcome. Finally, we did not collect more information concerning the participants’ lifestyle characteristics. Analyzing these relevant data may aid in further understanding our findings.

In summary, the current work demonstrated a plausible relationship between low vitamin D, functional network dysconnectivity, and clinical presentations in female patients with MDD. Our data provide evidence for a female-specific neurobiological mechanism whereby low vitamin D might give rise to MDD and its associated clinical characteristics. More broadly, these findings may inform a novel conceptualization that adjuvant vitamin D supplementation therapy may yield clinical benefits in improving treatment outcomes in female patients with MDD.

## Data Availability Statement

The raw data supporting the conclusions of this article will be made available by the authors, without undue reservation.

## Ethics Statement

The studies involving human participants were reviewed and approved by The First Affiliated Hospital of Anhui Medical University. The patients/participants provided their written informed consent to participate in this study.

## Author Contributions

WZ and YY designed the research, analyzed the data, and wrote the manuscript. D-MZ conducted the clinical evaluation and acquired the clinical data. SC, PJ, CZ, and YZ acquired the MRI data. JZ reviewed the manuscript for intellectual content. All authors contributed to and have approved the final manuscript.

## Conflict of Interest

The authors declare that the research was conducted in the absence of any commercial or financial relationships that could be construed as a potential conflict of interest.

## Publisher’s Note

All claims expressed in this article are solely those of the authors and do not necessarily represent those of their affiliated organizations, or those of the publisher, the editors and the reviewers. Any product that may be evaluated in this article, or claim that may be made by its manufacturer, is not guaranteed or endorsed by the publisher.

## Footnotes

1
http://www.fil.ion.ucl.ac.uk/spm

2
http://rfmri.org/dpabi

3
mialab.mrn.org/software/gift/

4
http://research.ics.tkk.fi/ica/icasso/

5
http://www.processmacro.org/
