# Supplementary material for: The Relationship Between Vitamin D, Clinical Manifestations, and Functional Network Connectivity in Female Patients With Major Depressive Disorder
Source: Front Aging Neurosci. 2022 Feb 10;14:817607. doi: 10.3389/fnagi.2022.817607 (PMC8867179; doi:10.3389/fnagi.2022.817607)
Supplement: Supplementary file 1 [file Data_Sheet_1.doc]

**Supplementary Materials**

**Table S1.** Demographic and clinical characteristics of all participants

| Characteristics | MDD (122) | HC (119) | Statistics | *P* value |
| --- | --- | --- | --- | --- |
| Gender (female/male) | 82/40 | 82/37 | χ^2^ = 0.080 | 0.778 |
| Age (years) | 42.77 ± 11.50  (18 - 62) | 43.81 ± 13.85  (21 - 65) | *t* = -0.641 | 0.522 |
| Education (years) | 8.86 ± 3.64  (0 - 16) | 11.52 ± 4.69  (0 - 20) | *t* = -4.902 | < 0.001 |
| BMI (kg/m^2^ ) | 22.91 ± 3.80  (13.93 - 34.60) | 23.23 ± 2.76  (15.98 - 31.56) | *t* = -0.752 | 0.453 |
| HAMD | 28.87 ± 11.31  (1 - 55) | 1.13 ± 2.54  (0 - 19) | *t* = 26.418 | < 0.001 |
| HAMA | 19.88 ± 7.38  (2 - 35) | 1.41 ± 2.86  (0 - 21) | *t* = 25.729 | < 0.001 |
| EBPM | 2.66 ± 2.81  (0 - 8) | 5.71 ± 2.71  (0 - 8) | *t* = -8.561 | < 0.001 |
| TBPM | 2.17 ± 2.24  (0 - 6) | 5.37 ± 1.35  (0 - 6) | *t* = -13.435 | < 0.001 |
| CPT-IP-2 | 2.23 ± 1.03  (-0.12 - 4.24) | 3.14 ± 0.97  (0.27 - 4.24) | *t* = -7.087 | < 0.001 |
| CPT-IP-3 | 1.68 ± 0.95  (-0.26 - 4.24) | 2.38 ± 1.13  (-0.11 - 4.24) | *t* = -5.268 | < 0.001 |
| CPT-IP-4 | 0.93 ± 0.73  (-0.41 - 3.12) | 1.34 ± 0.87  (-0.44 - 3.62) | *t* = -3.933 | < 0.001 |
| SCVD (nmol/L) | 42.62 ± 14.38  (15.95 - 96.34) | 52.70 ± 16.92  (21.50 - 105.75) | *t* = -4.986 | < 0.001 |
| FD (mm) | 0.13 ± 0.09  (0.04 - 0.60) | 0.14 ± 0.08  (0.05 - 0.40) | *t* = -0.995 | 0.321 |
| Illness duration (months) | 63.18 ± 71.33  (0.30 - 306) | - | - | - |
| Onset age (years) | 37.16 ± 11. 29  (12 - 55) | - | - | - |
| Episode number | 2.53 ± 2.23  (1 - 21) | - | - | - |
| Antidepressant medications |  |  |  |  |
| SSRIs | 82 | - | - | - |
| SNRIs | 33 | - | - | - |
| NaSSA | 7 | - | - | - |

Except for gender designation, data are expressed as means ± standard deviations. Numbers in parentheses are the range. Abbreviations: MDD, major depressive disorder; HC, healthy controls; BMI, body mass index; HAMD, Hamilton Rating Scale for Depression; HAMA, Hamilton Rating Scale for Anxiety; EBPM, event-based prospective memory; TBPM, time-based prospective memory; CPT-IP, Continuous Performance Task-Identical Pairs; SCVD, serum concentration of vitamin D; FD, frame-wise displacement; SSRIs, selective serotonin reuptake inhibitors; SNRIs, serotonin norepinephrine reuptake inhibitors; NaSSA, noradrenergic and specific serotonergic antidepressant.

**Table S2.** Correlations between SCVD and clinical variables in females

| Clinical variables | SCVD (nmol/L) | |
| --- | --- | --- |
|  | *pr* | *P* |
| EBPM | 0.267 | < 0.001^#^ |
| TBPM | 0.355 | < 0.001^#^ |
| CPT-IP-2 | 0.215 | 0.006^#^ |
| CPT-IP-3 | 0.121 | 0.126 |
| CPT-IP-4 | 0.087 | 0.273 |
| HAMD^*^ | -0.074 | 0.516 |
| HAMA^*^ | -0.071 | 0.529 |

Abbreviations: SCVD, serum concentration of vitamin D; EBPM, event-based prospective memory; TBPM, time-based prospective memory; CPT-IP, Continuous Performance Task-Identical Pairs; HAMD, Hamilton Rating Scale for Depression; HAMA, Hamilton Rating Scale for Anxiety; *pr*, partial correlation coefficient.

^#^means *P* < 0.05 with false discovery rate correction for multiple comparisons.

^*^ Correlation analyses were performed in 82 female patients with major depressive disorder.

**Table S3.** Correlations between functional network connectivity and clinical variables in females

| Functional network connectivity | EBPM | TBPM | CPT-IP-2 | CPT-IP-3 | CPT-IP-4 | HAMD^*^ | HAMA^*^ |
| --- | --- | --- | --- | --- | --- | --- | --- |
| Right aANG | -0.194^#^ | -0.234^#^ | -0.095 | 0.035 | 0.100 | -0.045 | 0.025 |
| Right pANG | -0.154 | -0.195^#^ | -0.066 | -0.001 | 0.106 | 0.125 | 0.166 |
| Right LPC | -0.288^#^ | -0.334^#^ | -0.281^#^ | -0.046 | 0.041 | -0.150 | -0.097 |
| Right CAL | -0.234^#^ | -0.160 | -0.136 | -0.102 | -0.056 | -0.098 | -0.049 |
| Right IPG | -0.188^#^ | -0.244^#^ | -0.118 | -0.061 | -0.052 | -0.126 | -0.104 |
| Left MCC | -0.126 | -0.148 | -0.125 | -0.132 | -0.103 | -0.052 | -0.051 |
| VAN-SMN | -0.148 | -0.165 | -0.221^#^ | -0.047 | 0.011 | -0.231 | -0.232 |

Abbreviations: aANG, anterior angular gyrus; pANG, posterior angular gyrus; LPC, lateral parietal cortex; CAL, calcarine sulcus; IPG, inferior parietal gyrus; MCC, middle cingulate cortex; VAN, ventral attention network; SMN, sensorimotor network; EBPM, event-based prospective memory; TBPM, time-based prospective memory; CPT-IP, Continuous Performance Task-Identical Pairs; HAMD, Hamilton Rating Scale for Depression; HAMA, Hamilton Rating Scale for Anxiety.

^#^means *P* < 0.05 with false discovery rate correction for multiple comparisons.

^*^ Correlation analyses were performed in 82 female patients with major depressive disorder.

**Table S4.** Correlations between SCVD and clinical variables in females after additionally controlling for BMI

| Clinical variables | SCVD (nmol/L) | |
| --- | --- | --- |
|  | *pr* | *P* |
| EBPM | 0.267 | < 0.001^#^ |
| TBPM | 0.355 | < 0.001^#^ |
| CPT-IP-2 | 0.215 | 0.006^#^ |
| CPT-IP-3 | 0.121 | 0.127 |
| CPT-IP-4 | 0.087 | 0.272 |
| HAMD^*^ | -0.080 | 0.486 |
| HAMA^*^ | -0.079 | 0.490 |

Abbreviations: SCVD, serum concentration of vitamin D; BMI, body mass index; EBPM, event-based prospective memory; TBPM, time-based prospective memory; CPT-IP, Continuous Performance Task-Identical Pairs; HAMD, Hamilton Rating Scale for Depression; HAMA, Hamilton Rating Scale for Anxiety; *pr*, partial correlation coefficient.

^#^means *P* < 0.05 with false discovery rate correction for multiple comparisons.

^*^ Correlation analyses were performed in 82 female patients with major depressive disorder.

**Table S5.** Correlations between SCVD and functional network connectivity in females after additionally controlling for BMI

| Functional network connectivity | SCVD (nmol/L) | |
| --- | --- | --- |
|  | *pr* | *P* |
| Right aANG | -0.257 | 0.001^#^ |
| Right pANG | -0.184 | 0.020^#^ |
| Right LPC | -0.276 | < 0.001^#^ |
| Right CAL | -0.168 | 0.034^#^ |
| Right IPG | -0.180 | 0.023^#^ |
| Left MCC | -0.183 | 0.020^#^ |
| VAN-SMN | -0.273 | < 0.001^#^ |

Abbreviations: SCVD, serum concentration of vitamin D; BMI, body mass index; aANG, anterior angular gyrus; pANG, posterior angular gyrus; LPC, lateral parietal cortex; CAL, calcarine sulcus; IPG, inferior parietal gyrus; MCC, middle cingulate cortex; VAN, ventral attention network; SMN, sensorimotor network; *pr*, partial correlation coefficient.

^#^means *P* < 0.05 with false discovery rate correction for multiple comparisons.

**Table S6.** Correlations between SCVD and clinical variables in males

| Clinical variables | SCVD (nmol/L) | |
| --- | --- | --- |
|  | *pr* | *P* |
| EBPM | -0.008 | 0.946 |
| TBPM | 0.133 | 0.256 |
| CPT-IP-2 | 0.048 | 0.684 |
| CPT-IP-3 | 0.034 | 0.769 |
| CPT-IP-4 | 0.208 | 0.073 |
| HAMD^*^ | -0.092 | 0.581 |
| HAMA^*^ | -0.043 | 0.799 |

Abbreviations: SCVD, serum concentration of vitamin D; EBPM, event-based prospective memory; TBPM, time-based prospective memory; CPT-IP, Continuous Performance Task-Identical Pairs; HAMD, Hamilton Rating Scale for Depression; HAMA, Hamilton Rating Scale for Anxiety; *pr*, partial correlation coefficient.

^*^ Correlation analyses were performed in 40 male patients with major depressive disorder.

**Table S7.** Correlations between SCVD and functional network connectivity in males

| Functional network connectivity | SCVD (nmol/L) | |
| --- | --- | --- |
|  | *pr* | *P* |
| Right aANG | -0.057 | 0.629 |
| Right pANG | -0.001 | 0.995 |
| Right LPC | 0.061 | 0.603 |
| Right CAL | 0.237 | 0.042 |
| Right IPG | -0.101 | 0.392 |
| Left MCC | -0.090 | 0.446 |
| VAN-SMN | 0.341 | 0.003 |

Abbreviations: SCVD, serum concentration of vitamin D; aANG, anterior angular gyrus; pANG, posterior angular gyrus; LPC, lateral parietal cortex; CAL, calcarine sulcus; IPG, inferior parietal gyrus; MCC, middle cingulate cortex; VAN, ventral attention network; SMN, sensorimotor network; *pr*, partial correlation coefficient.

**Table S8.** Correlations between functional network connectivity and clinical variables in males

| Functional network connectivity | EBPM | | TBPM | | CPT-IP-2 | | CPT-IP-3 | | CPT-IP-4 | | HAMD^*^ | | HAMA^*^ | |  |
| --- | --- | --- | --- | --- | --- | --- | --- | --- | --- | --- | --- | --- | --- | --- | --- |
| Right aANG | | -0.142 | | -0.252 | | -0.227 | | 0.042 | | 0.093 | | 0.177 | | 0.123 | |
| Right pANG | | -0.121 | | -0.067 | | 0.007 | | 0.062 | | 0.082 | | 0.170 | | 0.191 | |
| Right LPC | | -0.334^#^ | | -0.416^#^ | | -0.315^#^ | | -0.046 | | -0.064 | | 0.290 | | 0.201 | |
| Right CAL | | -0.081 | | -0.113 | | -0.054 | | -0.029 | | -0.104 | | 0.042 | | 0.174 | |
| Right IPG | | 0.060 | | 0.005 | | 0.052 | | -0.001 | | -0.042 | | 0.269 | | 0.096 | |
| Left MCC | | 0.002 | | -0.129 | | < 0.001 | | 0.072 | | 0.071 | | 0.096 | | 0.074 | |
| VAN-SMN | | -0.018 | | -0.096 | | 0.095 | | 0.158 | | 0.063 | | 0.070 | | -0.037 | |

Abbreviations: aANG, anterior angular gyrus; pANG, posterior angular gyrus; LPC, lateral parietal cortex; CAL, calcarine sulcus; IPG, inferior parietal gyrus; MCC, middle cingulate cortex; VAN, ventral attention network; SMN, sensorimotor network; EBPM, event-based prospective memory; TBPM, time-based prospective memory; CPT-IP, Continuous Performance Task-Identical Pairs; HAMD, Hamilton Rating Scale for Depression; HAMA, Hamilton Rating Scale for Anxiety.

^#^means *P* < 0.05 with false discovery rate correction for multiple comparisons.

^*^ Correlation analyses were performed in 40 male patients with major depressive disorder.


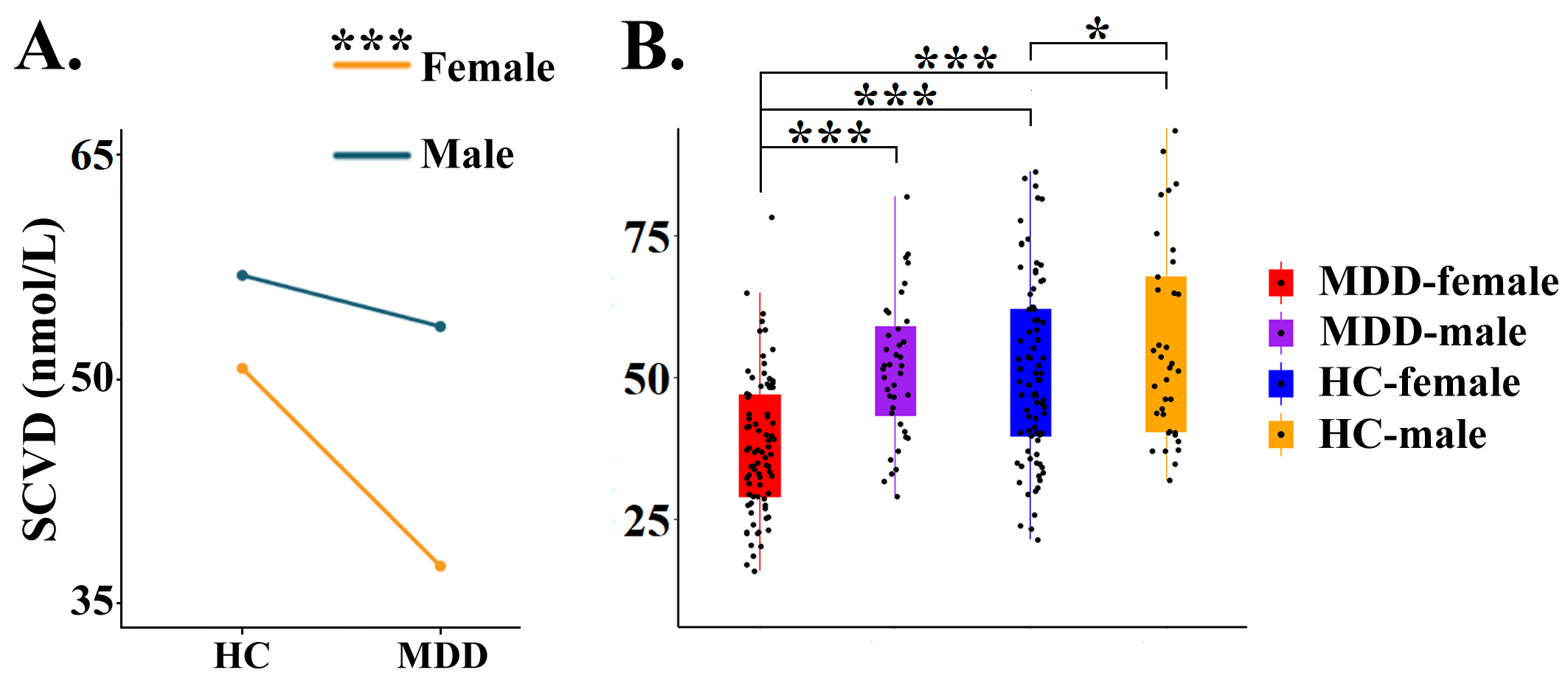


**Figure S1.** Interaction effect of group × gender on SCVD. (A) and (B) Significant group differences in females but not males. ^*^ *P* < 0.05; ^***^ *P* < 0.001. Abbreviations: SCVD, serum concentration of vitamin D; MDD, major depressive disorder; HC, healthy controls.


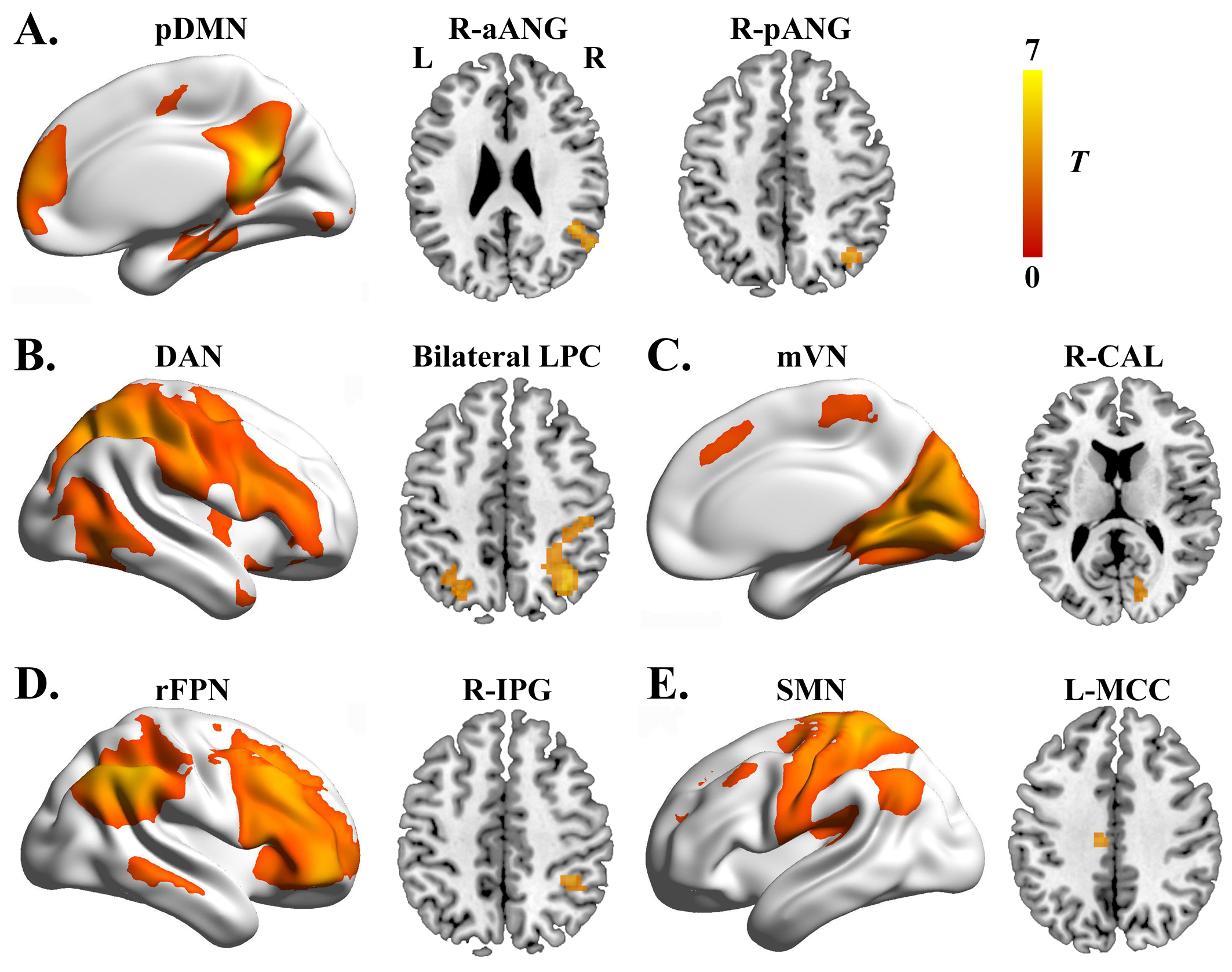


**Figure S2.** Brain regions showing increased intranetwork functional connectivity in female MDD patients relative to female HC. Abbreviations: pDMN, posterior default mode network; aANG, anterior angular gyrus; pANG, posterior angular gyrus; DAN, dorsal attention network; LPC, lateral parietal cortex; mVN, medial visual network; CAL, calcarine sulcus; rFPN, right frontoparietal network; IPG, inferior parietal gyrus; SMN, sensorimotor network; MCC, middle cingulate cortex; MDD, major depressive disorder; HC, healthy controls; L, left; R, right.
